# Adenovirus-like inclusion body hepatitis in a flock of broiler chickens in Kermanshah province, Iran

**Published:** 2015-03-15

**Authors:** Morad Rahimi, Zahra Minoosh Siavosh Haghighi

**Affiliations:** 1 *Department of Clinical Sciences, Faculty of Veterinary Medicine, Razi University, Kermanshah, Iran; *; 2 *Department of Pathobiology, Faculty of Veterinary Medicine, Razi University, Kermanshah, Iran.*

**Keywords:** Adenovirus, Broiler, Hepatitis, Inclusion body, Iran

## Abstract

Inclusion body hepatitis (IBH) has been reported in many countries in the world. The IBH or similar cases characterized by hepatitis and presence of intra-nuclear inclusion bodies in hepatocytes have not been reported in broiler chickens in Iran. This is the first report on outbreak of adenovirus-like inclusion body hepatitis in Iran. On October 2012, an onset of high acute mortality in a flock of 2 day-old broiler chickens was reported to the Veterinary Clinic, Faculty of Veterinary Medicine, Razi University, Kermanshah, Iran. The birds showed lethargy, huddling, ruffled feathers, and inappetence. At necropsy the livers were the primary organ affected which were enlarged, pale yellow with necrotic foci and multiple petechial hemorrhages. Tissue samples of liver, kidneys and heart were fixed in 10% buffered formalin. They were processed and stained with hematoxylin and eosin for histopathological studies. Significant microscopic lesions were seen in the livers. Large eosinophilic intra-nuclear inclusion bodies were seen in hepatocytes. Based on the acute high mortality, age of the broilers, gross lesions and histopathological findings (especially intra-nuclear inclusion bodies), the condition was diagnosed as adenovirus-like inclusion body hepatitis.

## Introduction

Inclusion body hepatitis (IBH) was recognized for the first time in the USA by Helmboldt and Frazier.^1^ Since then, the disease has been reported in many countries in the world. The IBH is caused by several serotypes of fowl adenovirus.^2^^,^^3^ Fowl adenovirus are resistant to several disinfectants, heat and pH changes.^4^ Although IBH is transmitted by both vertical and horizontal means, vertical transmission is reported as a very efficient way to spread from parent birds to progenies.^5^^,^^6^ Horizontal transmission of the causative virus occurs by the oral-fecal route and further spread takes place by mechanical means and by contamination with infected feces.^4^

Inclusion body hepatitis normally occurs in meat-type chickens at 3 to 7 weeks of age, but it has been reported in birds as young as 7 day-old and as old as 20 weeks. ^7^^,^^8^ In natural outbreaks, the disease is characterized by sudden onset of mortality ranging from 2 to 40 percent in chickens. High mortality occurs when the affected birds are less than three weeks of age. Depending on the pathogenicity of the virus, immune status of the chicks and concurrent secondary infections, mortality up to 80% has been reported. Generally, mortality peaks within three to four days and declines within 9-14 days. Clinically, affected birds show lethargy, huddling, ruffled feathers, and inappetence.^4^ Gross lesions of IBH include an enlarged pale and friable liver sometimes with necrotic foci. Ecchymotic hemorrhages may be also seen in the liver and less consistently in leg and breast muscles. In most cases, the main lesions are in the liver.^5^^,^^9^^,^^10^

The laboratory diagnosis of adenovirus infections, including IBH in chickens is in most cases based on histological investigations and detection of intra-nuclear inclusion bodies in hepatocytes or detection of the antigen or virus particles using immunofluorescence test or electron microscopy.^4^ Histopathological lesions include areas of focal necrosis and some of the hepatocytes have intra-nuclear inclusion bodies. Inclusion bodies can be eosinophilic, large, round, or irregularly shaped with a clear pale halo or occasionally basophilic.^11^^-^^13^ Sub-epicardial hemorrhages with multifocal necrosis in the myocardium have been the major reported findings in the heart.^14^^,^^15^

According to our current knowledge, IBH or similar cases characterized by hepatitis and presence of intra-nuclear inclusion bodies in hepatocytes have not been reported in broiler chickens in Iran. Therefore, this outbreak of adenovirus-like inclusion body hepatitis in a broiler farm in Iran is reported here for the first time.

## Case Description

On October 2012, an onset of high acute mortality in a flock of 2-day old broiler chickens was reported to Veterinary Clinic, Faculty of Veterinary Medicine, Razi University, Kermanshah, Iran. During the disease outbreak, the farm was visited regularly. The farm was located in northwest of Kermanshah province. Totally, 35,000 broiler chickens were kept in 5 windowless houses (7000 birds per house). Wood shavings were used as litter. All houses were equipped with central heaters, pad cooling systems and ventilators to provide the optimum environmental conditions for the broiler chickens. The birds were fed with a commercial corn-soybean meal starter diet. Feed and water were provided *ad libitum*. The lighting program was 23 hr light and 1 hr dark. All chickens of the farm were vaccinated against infectious bronchitis virus at the time of arrival to the farm. 

At farm visits, clinical signs and daily mortality were recorded. Clinically, the birds showed lethargy, ruffled feathers, and inappetence. Huddling and smothering were the major observed clinical signs. Mortality of the disease started with 300 chicks per 24 hr on 2^nd^ day of age, peaked at 410 on 5^th^ day and then gradually declined to normal at the age of 21 days. During the course of disease, total mortality reached about 14%.

On daily visits, a complete necropsy was carried out on at least five newly dead or euthanized sick birds of each house. Tissue samples of liver, kidneys and heart were fixed in 10% buffered formalin. They were processed according to routine procedures and stained with hematoxylin and eosin (H & E) for histopathology. 

**Fig. 1:**
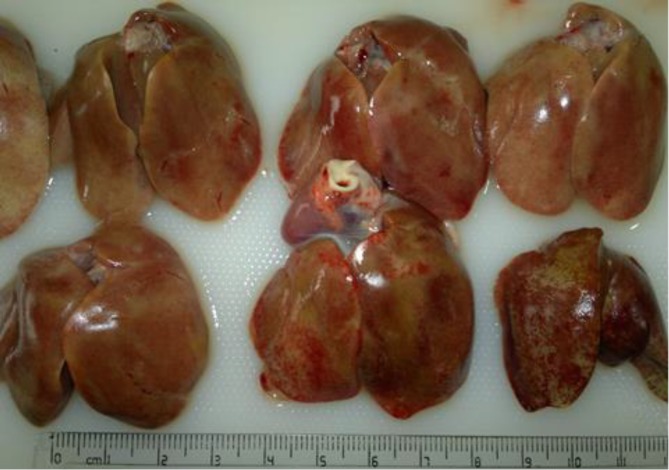
Chicken livers affected by adenovirus-like inclusion body hepatitis; they were enlarged, pale yellow and friable with necrotic foci and petechial hemorrhages.

Due to some restrictions, we could not follow up the case with virus isolation or molecular methods to identify the exact etiological agent of the disease. Therefore, based on the acute high mortality, age of the broilers, and gross and microscopic lesions (especially intra-nuclear inclusion bodies), the condition was diagnosed as adenovirus-like inclusion body hepatitis. At necropsy the livers were the primary organ affected which were enlarged, pale yellow with necrotic foci and multiple petechial hemorrhages (Fig. 1).

**Fig. 2:**
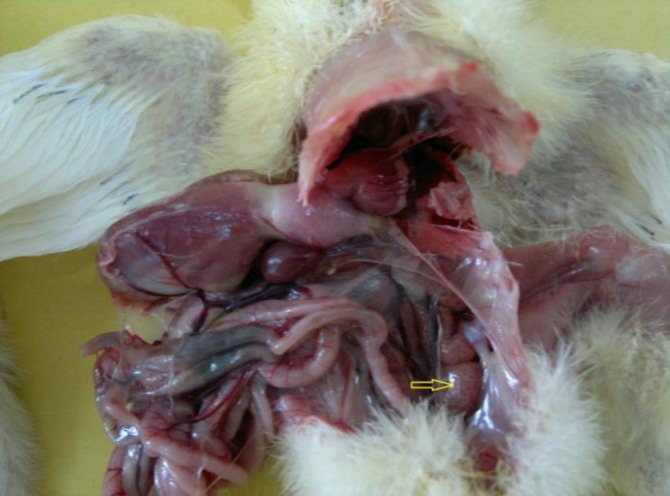
Swelling (Arrow) and urates deposition in the kidneys of a broiler chicken with adenovirus-like inclusion body hepatitis.

**Fig. 3:**
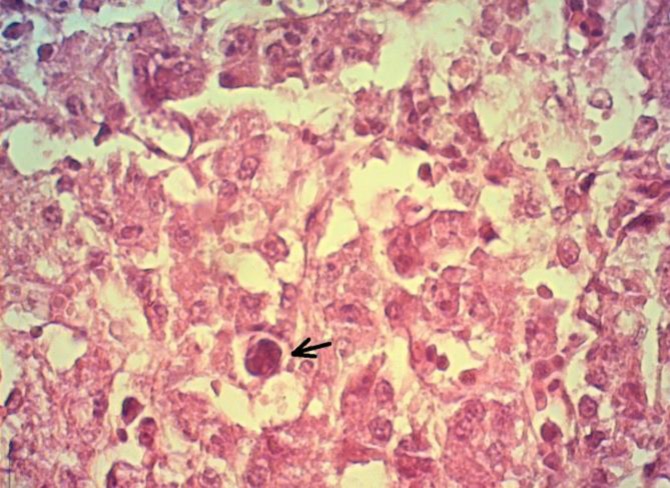
Liver, large eosinophilic intra-nuclear inclusion body (Arrow), (H & E, 1000×).

Kidneys appeared swollen and pale (Fig. 2). On histopathological examinations, significant lesions were seen in the liver. Focal hepatitis with infiltration of mononuclear inflammatory cells was noted. Large eosinophilic intra-nuclear inclusion bodies were seen in hepatocytes (Fig. 3). Kidneys showed severe hyperemia, tubular epithelial cells degeneration, intra-tubular cellular cast formation, and mild interstitial nephritis. Mild focal myocarditis, with degenerated muscle fibers was seen in the heart sections.

## Discussion

In this study, high acute mortality started at 2 day-old broiler chickens, the mortality peaked on 5^th^ day and gradually declined to normal at the age of 21 days. The IBH can affect all ages of broilers and all chickens are found to be susceptible even in immunological intact chicks during the first 2 to 3 weeks of life. Chicks as young as five days of age can develop IBH signs and lesions. There is a clear age effect with avian adenoviruses, as the age of the host increases, the degree of multiplication of the viruses within the host is restricted^16^ and the mortality they produce is reduced.^17^

Some of the clinical signs and lesions of the disease reported here were similar to those of hydropericardium syndrome (HPS; Angara disease) reported by Ahmad *et al*. and Anjum *et al*. ^14^^,^^18^ However, the course of the disease, mortality rate and other lesions were different. It started at two days of age, the mortality rate (14%) was lower than that of HPS, and there was no accumulation of straw colored fluid in the pericardial sac. Typically, the mortality of HPS starts at three weeks of age, and it causes between 20 and 80% mortality.^18^^,^^19^ The main pathological findings of HPS are the accumulation of a clear, straw colored fluid in the pericardial sac.^20^

We could not follow up the case with virus isolation or molecular methods to identify the exact etiological agent of the disease. Therefore, based on the acute high mortality, age of the broilers, and gross and microscopic lesions (especially intra-nuclear inclusion bodies), the condition was diagnosed as adenovirus-like inclusion body hepatitis. Virus isolation can be performed on cloacal swabs (feces) and fresh liver with lesions. The isolation of an adenovirus from the appropriate organ does not necessarily mean it is the etiological agent of the disease. Such an isolate may be the causative agent, it may have been latent in the bird or it may have been a chance isolate. In assessing the potential of an isolate to cause disease the immune status of the chick must be considered.^5^ Pathognomonic intra-nuclear inclusion bodies in liver cells were the same as IBH of avian species. Hematoxylin and eosin staining of liver sections, and demonstration of intra-nuclear eosinophilic inclusions, may provide a nonspecific indication of the presence of DNA containing virus.

Further studies have to be done to assess the prevalence of serotypes of fowl adenoviruses and occurrence of IBH in poultry flocks in Iran. Demonstration of pathognomonic intra-nuclear inclusion bodies in hepatocytes indicates the involvement of a DNA containing virus. Serology can be used to monitor progression but obviously does not indicate active infection. Virus isolation and molecular methods can also be used for detection and typing of field isolates.
